# Parental coronavirus disease vaccine hesitancy for children in Bangladesh: a cross-sectional study

**DOI:** 10.12688/f1000research.76181.1

**Published:** 2022-01-25

**Authors:** Mohammad Ali, Sohel Ahmed, Atia Sharmin Bonna, Abu-sufian Sarkar, Md. Ariful Islam, Tania Akter Urmi, Tasnuva Samarukh Proma

**Affiliations:** 1 Department of Physiotherapy and Rehabilitation, Uttara Adhunik Medical College, Dhaka, 1230, Bangladesh; 2 Hasna Hena Pain Physiotherapy and Public Health Research Center, Uttara Model Town, Dhaka, 1230, Bangladesh; 3 Department of Physiotherapy, Mount Adora Hospital, Sylhel, 3100, Bangladesh; 4 Save the Children, Dhaka, 1212, Bangladesh; 5 Bashundhara Kings Football Club, Dhaka, 1229, Bangladesh; 6 Zaman Mordan Hospital, Sherpur, 2100, Bangladesh; 7 Jatio protibondhi seba o sahajjo kendro, Gopalgonj, 8100, Bangladesh; 8 Advanced Physiotherapy & Rehab Solution, Women's Children's & General Hospital, Dhaka, 1209, Bangladesh

**Keywords:** Bangladesh, COVID-19, developing countries, parents, pediatrics, vaccine hesitancy.

## Abstract

**Background** **:** Coronavirus disease 2019 (COVID-19) requires mass immunization to control the symptoms and global spread of severe acute respiratory syndrome coronavirus 2. Data from developed countries reported a high prevalence of parental COVID-19 vaccine hesitancy. However, parental vaccine hesitancy data in countries with low and middle income are scarce. The goal of this study was to assess the prevalence of parental vaccine hesitancy and identify subgroups with higher odds of vaccine hesitancy.

**Methods:** A cross-sectional study was conducted on parents of children aged <18 years from October 10, 2021, to October 31, 2021. Parents aged ≥18 years underwent face-to-face interviews in randomly selected places in Bangladesh using a vaccine hesitancy questionnaire. Predictors were identified using binary logistic regression analysis.

**Results:** Data from 2633 eligible parents were analyzed. Overall, 42.8% reported COVID-19 vaccine hesitancy for their youngest child. The final model suggested that children's age, parent's age, religion, occupation, monthly household income, permanent address, living location, the status of tobacco use, adherence with the regular government vaccination programs (other than COVID-19), perception about COVID-19 vaccine efficacy among Bangladeshi children, self-vaccination intention, report about family members' illness or death from COVID-19, and perceived COVID-19 threat were the independent predictors of parental vaccine hesitancy.

**Conclusions** **:** Vaccine hesitation varied based on sociodemographic characteristics, religion, behavior, and perceived COVID-19 threat. Therefore, interventions focused on addressing vaccine hesitancy among subgroups are warranted.

## Introduction

Mass immunization against coronavirus disease 2019 (COVID-19) is one of the gold standard measures to control the spread of symptomatic severe acute respiratory syndrome coronavirus 2 (SARS-CoV-2) and end the global pandemic. ^1^ Many countries have targeted vaccinating at least 70% of their total population to achieve herd immunity, including individuals aged ≤18 years. ^2^^,^^3^ However, vaccine hesitancy, which is defined as a delay in acceptance or refusal of vaccines despite the availability of vaccination services, ^4^ is a significant threat to the smooth uptake of vaccinations worldwide. ^5^

Since December 2019, >250 million COVID-19 cases have been identified globally, and >50 million people have died of this disease, with a significantly high prevalence in older adults. ^6^ However, the infection rate among children and adolescents is not negligible, and they can carry and spread the virus. ^7^ Furthermore, unvaccinated populations are supposedly suitable hosts for new variants. ^8^

Some recent data indicate that a few countries, for example, the USA are unlikely to reach the 70% target for herd immunity; however, vaccinating 22% of the American population, that is, the pediatric population, would boost community protection against COVID-19. ^3^ Nonetheless, more than one in three parents in the USA were vaccine-hesitant for their children. ^3^ In China, this rate was 52.5%. ^9^ Along with sociodemographic variabilities and religious beliefs, overall vaccine hesitancy has also varied by political theology, people's perceived pandemic threat, or the socioeconomic status of the target population. ^10^^–^^12^ Additionally, revealing adverse effects, the vaccine's effectiveness in children, and research availability among the child's age groups may play a crucial role when parents decide to vaccinate their children. Furthermore, a survey revealed that along with potential immediate adverse effects of the vaccines, the possible long-term harmful effects were a growing concern for parents. ^13^

The triumph of immunization programs, among others, relies on the vaccination of a wide proportion of pediatric and adult populations in low- and middle-income countries where variants of concern, such as the SARS-CoV-2 B.1.617.2 Delta variant, have been detected. ^14^^,^^15^ In Bangladesh, by November 2021, only 18% of the whole population was fully vaccinated against this disease. ^16^ However, approximately 35% of Bangladeshi are aged <18 years. ^17^ To achieve herd immunity, this young cohort should be included in the mass vaccination program. Thus, the Bangladesh government planned to vaccinate students aged between 12 and 17 years. Vaccination among the young student cohort started in cities, including Dhaka, to a limited extent from November 1, 2021. ^18^

There is limited information about vaccine hesitancy among parents of children aged <18 years. In Bangladesh, a previous study revealed that 32% of the study population aged >18 years refused to be vaccinated against COVID-19. ^10^ We hypothesized that parental vaccine hesitancy would not match the rate in the adult population. This study sought to (1) conduct a nationally representative assessment of parental vaccine hesitancy and (2) identify subgroups of parents with higher odds of vaccine hesitancy.

## Methods

### Ethics statements

The Institutional Review Board of Uttara Adhunik Medical College and Hospital approved this study (Approval number: UAMC-IRB-2021/09). Written informed consent both for participation and publication of data was obtained from all participants.

### Study design and participants

This cross-sectional study was conducted in Bangladesh from October 10, 2021, to October 31, 2021. A margin of 2% error, a confidence level of 95%, and a response distribution of 50% were used to calculate the sample size to target fathers/mothers of 80 million children and obtain a minimum sample size of 2401 participants. ^19^^,^^20^ Approximately 3000 parents aged ≥18 years with children aged <18 years who permanently live in Bangladesh were conveniently invited to participate in an individualized interview session using a previously employed vaccine hesitancy questionnaire. ^10^^,^^21^^,^^35^ We received data from 2703 parents, as a result of a 10% refusal rate. However, 36 parents did not answer all the questions, thus were excluded. We also excluded 34 data points for the contradicting answers to the question. These exclusions meant that, only 2633 respondents were included in the final analysis. ^35^

### Study questionnaire

In the first part of the questionnaire, participants were queried regarding parental vaccine hesitancy and perceived COVID-19 threat. First, parents were asked about the likelihood of vaccinating their youngest children. Parental vaccine hesitancy, the dependent variable and a vital outcome of the study, was measured using the question, “If a vaccine that would be effective against coronavirus disease among children was available, how likely are you to get your children vaccinated?” (response options: very likely, somewhat likely, not likely, and definitely not). Second, participants were asked two questions regarding the perceived COVID-19 threat: (1) “How likely is it that your children or a family member could get infected with coronavirus in the next year?” (response options: very likely, somewhat likely, not likely, and definitely not). (2) “How concerned are you that your children or a family member could get infected with coronavirus in the next year?” (response options: very concerned, concerned, slightly concerned, and not concerned at all).

The second part of the questionnaire included a wide array of sociodemographic questions for both children and parents. A set of structured questions assessed the child's health (healthy/disabled), age, and sex. Information on parents' sex, age, religion, current marital status, education, employment status, monthly household income (Bangladeshi taka), permanent address, and region of residence (north, south, and central zones in Bangladesh, including Dhaka), current residence type (own/rented/others), family type (nuclear or extended, number of children, current tobacco use status, religious practice habits, and political affiliation was collected. Additionally, parents were asked some additional COVID-19 vaccine-related questions: “Do you think the COVID-19 vaccine will be effective among Bangladeshi children?” (response options: no, yes, or skeptical), “Have you received or plan to receive the COVID-19 vaccine,” “Did you or your family member(s) test positive for COVID-19,” and “Have you lost any of your family member(s) to COVID-19?” The last three questions received dichotomous (yes or no) answers.

### Sampling technique and data collection

Data was collected from all eight divisions of Bangladesh. A dual-stage cluster sampling technique was used to include potential samples for this study. We randomly chose marketplaces, shopping malls, waiting rooms of large hospitals, diagnostic centers, bus and rail stations, and residences and processed them as a cluster in the first stage. To obtain data from the parents of a child with a disability, we also visited randomly selected centers for disabled children. The list of given data collection sites was collected from the divisions' websites. In the second stage, we chose participants conveniently. Data from only the father or mother of a child were taken from a family with children to avoid repeating data.

Eight teams of two persons were created. A team member read the questions aloud to the interviewees individually. Acceptable options were asked and recorded in the question paper. Subsequently, the answers were checked and confirmed by the second team member. The coinvestigator reviewed the data collection sheets for completeness, accuracy, and internal consistency and secured them with the principal investigator. Individual face-to-face interviews were conducted to ensure the participants' privacy. All participants were informed of the voluntary nature of their participation, and the interviews were conducted in Bangla.

### Statistical analysis

The crucial outcome of this study was vaccine hesitancy. We dichotomized the four responses to the vaccine hesitancy question as either a positive (very likely and somewhat likely) or a negative (not likely and definitely not) attitude towards the COVID-19 vaccine. The Fisher exact test was used for two nominal variables, and chi-square test was used for more than two nominal variables to assess vaccine hesitancy rates and draw comparisons between the groups. Binary logistic regression analyses were performed to identify the predictors of parental COVID-19 vaccine hesitancy and compute adjusted odds ratios (AORs) with a 95% confidence interval (CI). Factors significantly associated with vaccine hesitancy in the descriptive analysis were included in the regression model. A goodness-of-fit test for the adjusted logistic regression model was performed using the Hosmer-Lemeshow test. The significance level was set at p<0.05, and SPSS (version 22.0; IBM Corp; RRID: SCR_002865) was used to perform all data analyses.

## Results

### Parents and children's characteristics

Overall, 2633 parents aged 34.97±7.87 years (mean±standard deviation) were included in the analysis, with 52.8% (1390) being women. In total, 396 (15%) parents of children with a physical disability were included. Among the children, 1372 (52.1%) were boys, and 1206 (45.8%) were in the 0–4-year-old group. Most parents (653, 24.8%) were in the 31–35-year-old group. Overall, 2358 (89.4%) parents were Muslim, 1791 (68%) were a nuclear family member, 1075 (40.8%) had two children, 1022 (38.8%) had a low education level, 756 (28.7%) were homemakers, and 833 (31.6%) had a low-middle household income. Among all participants, 1528 (58%) were from the village, 1323 (50%) were living in the central zone, including Dhaka, 1695 (64.4%) were tobacco non-users, 1797 (68.2%) were regular religious practitioners, and 1032 (39.2%) were politically neutral respondents. A total of 177 (6.3%) parents did not adhere to the regular government vaccination programs other than COVID-19, and 1458 (55.4%) remained skeptical about COVID-19 vaccine effectiveness for Bangladeshi children. Furthermore, 722 (27.4%) parents were either not vaccinated or did not receive the COVID-19 vaccine; however, 752 (28.6%) parents reported that they or their family members tested positive for COVID-19, and 151 (5.7%) lost a loved one to COVID-19. Details of the responses to the questions regarding the likelihood of children or family members' infection by COVID-19 and the level of concern about children or family members' disease in the next year are shown in Table 1.

**Table 1.** Descriptive analysis: Sociodemographic characteristics, COVID-19 threat, and parental vaccine hesitancy.

Variables	Total sample n (%)	Likelihood of vaccinating children		P value
		Not likely/definitely not n (%)	Very likely/somewhat likely n (%)	
All participants	2633 (100)	1126 (42.8)	1507 (57.2)	N/A
*Children's health*				0.507
Healthy	2237 (85)	957 (42.8)	1280 (57.2)	
Disabled	396 (15)	169 (42.7)	227 (57.3)	
*Children's age group*				**0.000**
0–4	1206 (45.8)	649 (53.8)	557 (46.2)	
5–9	870 (33)	344 (39.5)	526 (60.5)	
10–14	354 (13.4)	98 (27.7)	256 (72.3)	
15–<18	203 (7.7)	35 (17.2)	168 (82.8)	
*Children's sex*				**0.000**
Male	1372 (52.1)	537 (39.1)	835 (60.9)	
Female	1261 (47.9)	589 (46.7)	672 (53.3)	
*Parents' age group*				**0.000**
18–25	268 (10.2)	146 (54.5)	122 (45.5)	
26–30	604 (22.9)	329 (54.5)	275 (45.5)	
31–35	653 (24.8)	288 (43.8)	367 (56.2)	
36–40	563 (21.4)	223 (39.6)	340 (60.4)	
41–45	285 (10.8)	86 (30.2)	199 (69.8)	
46–50	162 (6.2)	37 (22.8)	125 (77.2)	
≥ 51	98 (3.7)	19 (19.4)	79 (80.6)	
*Parents' sex*				0.237
Female	1390 (52.8)	604 (43.5)	786 (52.2)	
Male	1243 (47.2)	522 (42)	721 (58)	
*Marital status*				0.438
Married	2527 (96)	1082 (42.8)	1445 (57.2)	
Divorced or widowed	106 (4)	44 (41.5)	62 (58.5)	
*Religion*				**0.000**
Muslim	2358 (89.4)	1069 (45.4)	1285 (54.6)	
Hindu	258 (9.8)	56 (21.7)	202 (78.3)	
Buddhist	6 (.2)	0 (0)	6 (100)	
Christian	15 (.6)	1 (6.7)	14 (93.3)	
*Type of family*				0.167
Extended family	842 (32)	372 (44.2)	470 (55.8)	
Nuclear family	1791 (68)	754 (42.1)	1037 (57.9)	
*Number of children*				0.993
One	924 (35.1)	395 (42.7)	529 (57.3)	
Two	1075 (40.8)	461 (42.9)	614 (57.1)	
Three or more	634 (24.1)	270 (42.6)	364 (57.4)	
*Educational qualification*				**0.000**
≤ High school	1022 (38.8)	473 (46.3)	549 (53.7)	
College education	594 (22.6)	316 (53.2)	278 (46.8)	
Graduate	608 (23.1)	236 (38.8)	372 (61.2)	
Post-graduate	409 (15.5)	101 (24.7)	308 (75.3)	
*Occupation*				**0.000**
Service	677 (25.7)	248 (36.6)	429 (63.4)	
Business	472 (17.9)	198 (41.9)	274 (58.1)	
Unemployed	179 (6.8)	132 (73.7)	47 (26.3)	
Student	56 (2.1)	34 (30.7)	22 (39.3)	
Home maker	756 (28.7)	364 (48.1)	392 (51.9)	
Healthcare	216 (8.2)	64 (29.6)	152 (70.4)	
Daily labor	277 (10.5)	86 (31)	191 (69)	
*Monthly household income (৳)*				**0.000**
<৳15 000	799 (30.3)	401 (50.2)	398 (49.8)	
৳ 15000–30000	833 (31.6)	409 (49.1)	424 (50.9)	
৳ 31000–45000	433 (16.4)	150 (34.6)	283 (65.4)	
>৳ 45000	568 (21.6)	166 (29.2)	402 (70.8)	
*Current residence type*				**0.030**
Own	1436 (54.5)	646 (45)	790 (55)	
Rented	1075 (40.8)	427 (39.7)	648 (60.3)	
Others	122 (4.6)	53 (43.4)	69 (56.6)	
*Permanent address*				**0.000**
Village	1528 (58)	687 (45)	814 (55)	
Semi-urban	535 (20.3)	247 (45.2)	288 (53.8)	
City	570 (21.6)	192 (33.7)	378 (66.3)	
*Current living location*				**0.000**
Central zone	1323 (50.2)	542 (41.0)	781 (59.0)	
North zone	921 (35)	472 (51.2)	449 (48.8)	
South zone	389 (14.8)	112 (28.8)	277 (71.2)	
*Present tobacco user*				**0.000**
No	1695 (64.4)	679 (40.1)	1016 (59.9)	
Yes	938 (35.6)	447 (47.7)	491 (52.3)	
*Regular religious practice*				0.099
No	836 (31.8)	377 (45.1)	459 (54.9)	
Yes	1797 (68.2)	749 (41.7)	1048 (58.3)	
*Political affiliation*				**0.000**
Ruling party	779 (30.3)	283 (35.8)	516 (64.6)	
Opposition	296 (11.2)	175 (59.1)	121 (40.9)	
Neutral	1032 (39.2)	488 (47.3)	544 (52.7)	
Prefer not to say	506 (19.2)	180 (35.6)	326 (64.4)	
*Vaccinated/plan to vaccinate children under regular (other than COVID-19) govt. vaccination programs*				**0.000**
No	177 (6.7)	103 (58.2)	74 (41.8)	
Yes	2456 (93.3)	1023 (41.7)	1433 (58.3)	
*Do you think the COVID-19 vaccine will be effective in Bangladeshi children?*				**0.000**
No	167 (6.3)	151 (90.4)	16 (9.6)	
Yes	1008 (38.3)	54 (5.4)	954 (94.6)	
Skeptical	1458 (55.4)	921 (63.2)	537 (36.8)	
*Have you taken or plan to take the COVID-19 vaccine?*				**0.000**
No	722 (27.4)	653 (88)	87 (12)	
Yes	1911 (72.6)	491 (25.7)	1420 (74.3)	
*Have you or your family member(s) tested positive for COVID-19?*				**0.000**
No	1881 (71.4)	947 (50.3)	934 (49.7)	
Yes	752 (28.6)	179 (23.8)	573 (76.2)	
*Have you lost any of your family member(s) to COVID-19?*				**0.000**
No	2482 (94.3)	1105 (44.5)	1377 (55.5)	
Yes	151 (5.7)	21 (13.9)	130 (86.1)	
*Perceived likelihood of children or family members' infection in the next year*				**0.000**
Very likely	345 (13.1)	51 (14.8)	294 (85.2)	
Somewhat likely	1678 (63.7)	665 (39.6)	1013 (60.4)	
Not likely	451 (17.1)	296 (65.6)	155 (34.4)	
Definitely not	159 (6)	114 (71.7)	45 (28.3)	
*Level of concern about children or family members' infection in the next year*				**0.000**
Very concerned	386 (14.7)	72 (18.7)	314 (81.3)	
Concerned	1020 (38.7)	384 (37.6)	636 (62.4)	
Slightly concerned	673 (25.6)	303 (45)	370 (55)	
Not concerned at all	554 (21)	367 (66.2)	187 (33.8)	

### Results of descriptive analysis

**Figure 1.**
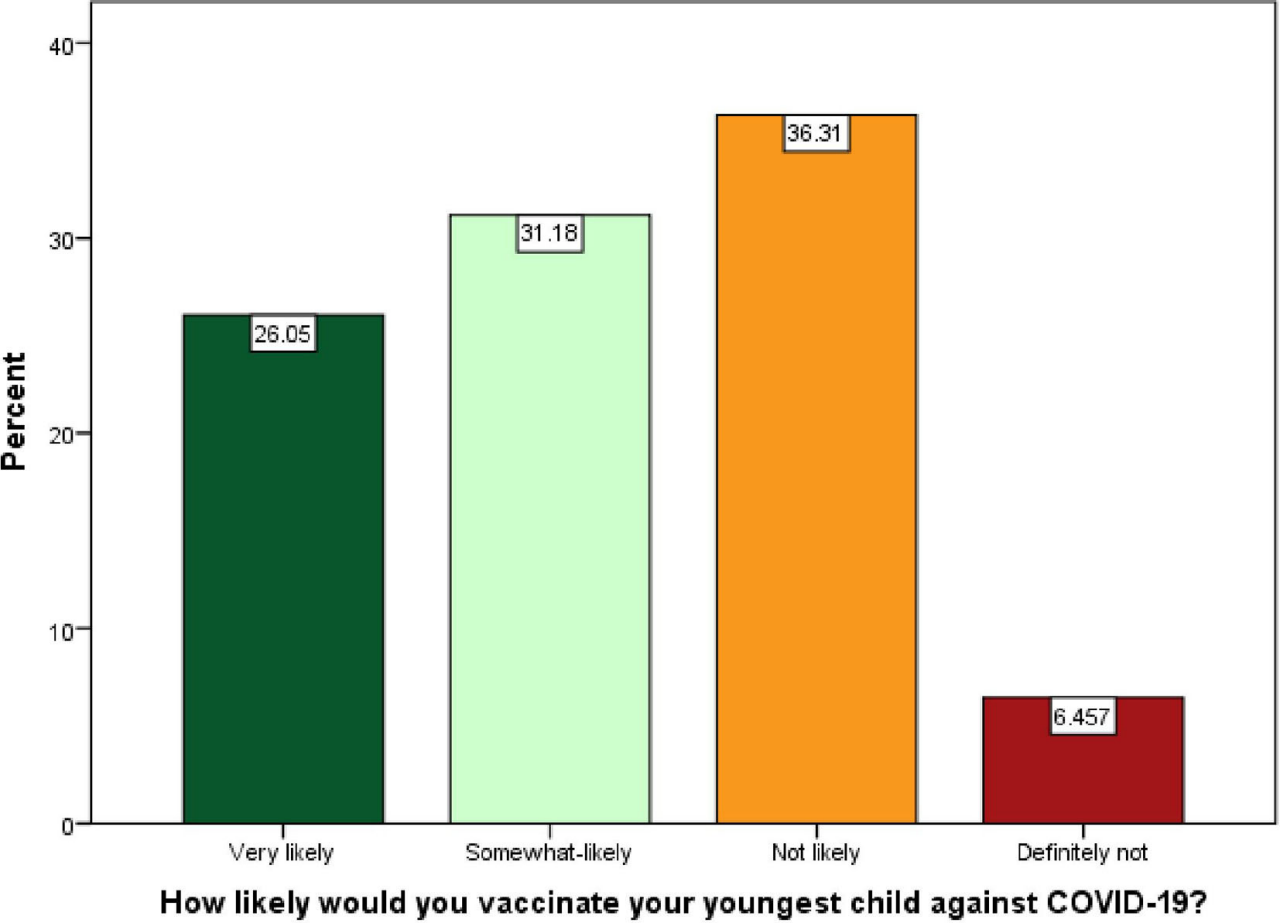
Likelihood of COVID-19 vaccine acceptance/refusal by Bangladeshi parents for children aged <18.

Overall, 42.8% of parents reported hesitancy towards the COVID-19 vaccine for their youngest child. Closer analysis revealed that 26.05% of parents were very likely, 31.18% were somewhat likely, and 36.31% were not likely to vaccinate their child. While only 6.46% were definitely not vaccinating their child against COVID-19 ( Figure 1). The incidence of vaccine hesitancy was significantly high among the parents of 0–4-year-old children (53.8%; p=0.000), parents of girls (46.7%; p=0.000), young parents (54.5%; p=0.000), Muslims (45.4%; p=0.000), parents who received college education (53.2%; p=0.000), unemployed parents (73.7%; p=0.000), parents with a household income of <৳15 000 (50.2%; p=0.000), those who lived in their own house (45%; p=0.030), came from a village (45%; p=0.000), lived in the north zone (51.2%; p=0.000), tobacco users (47.7%; p=0.000), and parents politically affiliated with opposition parties (59.1%; p=0.000). Similarly, participants who did not vaccinate or will not vaccinate their child with regular vaccines (other than COVID-19) available under government programs (58.2%; p=0.000), those who did not believe in the effectiveness of the COVID-19 vaccine for Bangladeshi children (90%; p=0.000), and those who did not or will not receive the COVID-19 vaccine for themselves (88%; p=0.000) showed high vaccine hesitancy. Parents who were not likely to believe that their children or a family member could be infected with COVID-19 in the next year (71.7%; p=0.000) and those not concerned about their children or a family member getting COVID-19 in the next year (66.2%; p=0.000) showed high levels vaccine hesitancy ( Table 1).

### Results of regression analysis

Subgroups with statistically significant higher odds of vaccine hesitancy were found to be parents of children aged 0–4 years (AOR=5.87, 95% CI=2.91–11.85; p=0.000), parents aged 26–30 years (AOR=2.73, 95% CI=1.04–7.16; p=0.035), Muslims (AOR=24.27, 95% CI=2.36–248.74; p=0.007), unemployed parents (AOR=2.94, 95% CI=1.35–6.41; p=0.007), parents with a household income of <৳15 000 (AOR=1.49, 95% CI=0.962–1.84; p=0.009), those from a semi-urban area (AOR=1.61, 95% CI=1.09–2.38; p=0.016), lived in the north zone (AOR=3.71, 95% CI=2.37–5.82; p=0.000), did not vaccinate or will not vaccinate their child with regular vaccines (other than COVID-19) available under government programs (AOR=1.93, 95% CI=1.19–3.14; p=0.007), did not believe in the effectiveness of COVID-19 vaccine for Bangladeshi children (AOR=5.80, 95% CI=3.12–10.78; p=0.000), and did not or will not receive the COVID-19 vaccine for themselves (AOR=10.15, 95% CI=7.16–14.39; p=0.000).

Contrarily, participants who were non-tobacco users (AOR=0.71, 95% CI=0.53–0.96; p=0.025), appeared to be very likely to believe that their children or a family member could be infected with COVID-19 in the next year (AOR=0.21, 95% CI=0.97–0.44; p=0.000), and very concerned about their children or a family member contracting COVID-19 in the next year (AOR=0.34, 95% CI=0.21–0.58; p=0.000) had significantly lower odds of COVID-19 vaccine hesitancy ( Table 2).

**Table 2.** Binary logistic regression: predictors of parental vaccine hesitancy in study participants.

Variables	Adjusted OR	Standard error	95% CI		P-value
*Children's age group (year)*					
0–4	5.876	0.358	2.914	11.850	**0.000**
5–10	2.845	0.348	1.438	5.631	**0.003**
11–14	1.101	0.359	0.545	2.225	0.789
15–<18	References				
*Children's sex*					
Male	0.918	0.127	0.716	1.176	0.498
Female	Reference				
*Parents' age group*					
18–25	1.513	0.522	0.544	4.205	0.427
26–30	2.732	0.492	1.042	7.165	**0.041**
31–35	2.755	0.481	1.073	7.075	**0.035**
36–40	2.737	0.476	1.077	6.958	**0.034**
41–45	2.524	0.487	0.972	6.552	0.057
46–50	1.101	0.522	0.396	3.064	0.853
≥ 51	Reference				
*Religion*					
Muslim	24.277	1.187	2.369	248.740	**0.007**
Hindu	18.704	1.206	1.758	198.977	**0.015**
Others	Reference				
*Educational qualification*					
≤ High school	1.002	0.279	0.579	1.731	0.995
College education	1.292	0.246	0.798	2.093	0.297
Graduate	1.007	0.223	0.651	1.560	0.974
Postgraduate	Reference				
*Occupation*					
Service	2.327	0.303	1.285	4.213	**0.005**
Business	1.463	0.310	0.797	2.686	0.219
Unemployed	2.943	0.398	1.350	6.415	**0.007**
Student	4.389	0.480	1.714	11.242	**0.002**
Home maker	2.105	0.281	1.214	3.650	**0.008**
Healthcare	2.775	0.370	1.344	5.728	**0.006**
Daily labor	Reference				
*Monthly household income (৳)*					
<৳15 000	1.499	0.266	0.962	1.840	**0.009**
৳ 15000–30000	0.826	0.210	0.547	1.248	0.364
৳ 31000–45000	0.559	0.216	0.366	0.854	**0.007**
>৳ 45000	Reference				
*Current residence type*					
Own	1.202	0.310	0.655	2.205	0.552
Rented	0.984	0.311	0.535	1.810	0.959
Others	Reference				
*Permanent address*					
Village	1.259	0.177	0.891	1.780	0.192
Semi-urban	1.614	0.198	1.095	2.381	**0.016**
City	Reference				
*Current living location*					
Central zone including Dhaka	3.112	0.213	2.049	4.727	**0.000**
North zone	3.716	0.230	2.370	5.827	**0.000**
South zone	Reference				
*Present tobacco user*					
No	0.716	0.149	0.535	0.959	**0.025**
Yes	Reference				
*Political affiliation*					
Ruling party	1.001	0.188	0.692	1.448	0.997
Opposition	1.310	0.261	0.785	2.188	0.301
Neutral	0.971	0.176	0.687	1.371	0.865
I prefer not to say	Reference				
*Vaccinated/plan to vaccinate children under regular (other than COVID-19) govt. vaccination programs*					
No	1.937	0.247	1.193	3.144	**0.007**
Yes	Reference				
*Do you think the COVID-19 vaccine will be effective for Bangladeshi children*					
No	5.805	0.316	3.124	10.786	**0.000**
Yes	0.052	0.171	0.037	0.073	**0.000**
Skeptical	Reference				
*Have you taken or plan to take the COVID-19 vaccine*					
No	10.152	0.178	7.161	14.392	**0.000**
Yes	Reference				
*Have you or your family member(s) tested positive for COVID-19*					
No	1.320	0.164	0.956	1.822	0.091
Yes	Reference				
*Have you lost any of your family member(s) to COVID-19*					
No	2.502	0.337	1.293	4.839	**0.006**
Yes	Reference				
*Perceived likelihood of children or family members' infection in the next year*					
Very likely	0.206	0.383	0.097	0.437	**0.000**
Somewhat likely	0.413	0.330	0.216	0.788	**0.007**
Not likely	0.687	0.329	0.360	1.309	.253
Definitely not	Reference				
*Level of concern about children or family members' infection in the next year*					
Very concerned	0.345	0.267	0.205	0.583	**0.000**
Concerned	0.502	0.207	0.334	0.754	**0.001**
Slightly concerned	0.593	0.202	0.399	0.881	**0.010**
Not concerned at all	Reference				

## Discussion

This nationally representative comprehensive study found a significant prevalence of COVID-19 vaccine hesitancy among parents in Bangladesh for their children. There were substantial differences in COVID-19 vaccine hesitancy according to sociodemographic factors and perceived COVID-19 threat among parents. The logistic regression model revealed that the children's age and parents' age, religion, occupation, monthly household income, permanent address, current living location, tobacco use, adherence to the regular government pediatric vaccination programs (other than COVID-19), perception about COVID-19 vaccine effectiveness for children in Bangladesh, self-vaccine hesitancy, loss of a family member due to COVID-19, and perceived COVID-19 threat can all be used independently to predict parental vaccine hesitancy for children aged <18 years.

To the best of our knowledge, this is the first study to examine parental COVID-19 vaccine hesitancy in Bangladesh. Furthermore, very limited data are available for parental vaccine hesitancy in Indian subcontinental countries. Thus, there is limited information about the previous hesitancy rate in this region. However, the rate observed in this study (42.8%) is similar to that found in the USA (42%) ^21^ and slightly lower than that found in China (52%). ^9^ Contrarily, the observed rate was significantly higher than that in Brazil, Malaysia, and Saudi Arabia. ^8^^,^^22^^,^^23^ The high parental vaccine hesitancy rate in Bangladesh induces further concern when vaccinating an optimum number of the subcontinental population.

Health behavior theory is centrally influenced by disease risk perception. Herein, a strong association was found between perceived COVID-19 threat and parental vaccine hesitancy. Parents who thought their children or family members were not likely to be at risk of contracting COVID-19 were highly hesitant toward vaccinate their children. Similarly, parents who were not concerned about children or family members’ infections were hesitant. Furthermore, vaccine hesitancy was significantly higher among those who did not believe or remain skeptical about COVID-19 vaccine efficacy among Bangladeshi children than those who did. These findings were consistent with the results of previous studies that measured COVID-19 vaccine hesitancy among the adult population. ^10^^,^^24^^,^^25^ Ignorance, belief in conspiracy theories, and even denial of the existence of COVID-19 may influence one's thought of self-vaccination or vaccinating a child. ^26^ Therefore, further studies are warranted to understand the in-depth association between COVID-19 threat and vaccine hesitancy among different population groups.

Parents who reported unemployment, an education level lower than or equal to high school, a household income of <৳15 000-৳ 30 000, and those from the village or semi-urban area were significantly more vaccine-hesitant. Similarly, a previous study found high vaccine hesitancy among unemployed, low education level, and lower-income groups of parents in several high- and middle-income countries. ^3^^,^^8^^,^^22^^,^^27^ Furthermore, our previous study on the adult Bangladeshi population found a similar scenario. ^10^ Global research and studies conducted in the USA and Saudi Arabia among the general population reported identical results. ^28^^–^^30^

A previous study found a higher prevalence of vaccine hesitancy among younger parents and parents of children aged between 0 and 4 years, ^21^ and our analysis yielded similar results. However, unlike a previous study in Malaysia (a multi-ethnic country), ^23^ we found high vaccine hesitancy among Muslim parents compared to non-Muslim parents in Bangladesh. Hence, more studies are warranted to understand the influence of religion on the decisions regarding vaccine acceptance and rejection.

Our study found a significantly high prevalence of parental vaccine hesitancy among tobacco users. A previous study also found a similar result, citing the association between unhealthy life practices and vaccine hesitancy among tobacco users. ^10^ Interestingly, vaccine hesitancy among parents living in the north zone of Bangladesh has also been shown to be high. This is likely because the north zone of Bangladesh is a tobacco-producing area with a high poverty level. Therefore, this information may explain the high prevalence of vaccine hesitancy among parents who are tobacco users and living in the north zone of Bangladesh.

Additionally, this study found a strong association between self-vaccination intention and vaccination decision for children. The incidence of parental vaccine hesitancy was 10 times higher among parents who did not receive or will not receive the COVID-19 vaccine for themselves than among those who did and will receive the COVID-19 vaccine. Furthermore, parents who did not get their children vaccinated with regular vaccines other than COVID-19 were also highly hesitant toward the COVID-19 vaccine for their children, indicating stubborn vaccine hesitancy among groups of people. Therefore, special advocacy targeting these groups is recommended when including them in the vaccination program to eradicate vaccine-preventable diseases. Conversely, we found high vaccination willingness among parents who reported that their family member(s) had either tested positive for or died of COVID-19. The harmful effect of COVID-19 may encourage patients to make a favorable decision about vaccinating their children when it is available.

### Strengths and limitations

This is the first study to reveal the rate of parental vaccine hesitancy for children in Bangladesh. This study included parents' data from all eight divisions of Bangladesh by randomly selected data collection sites; 52% women, 10% Hindu parents, and 1% other non-Muslim parents participated, providing a good representation of the population. Additionally, we conducted anonymous face-to-face interviews to reduce social desirability bias, minimize non-response, and maximize the quality of collected data. Data from parents of children with disabilities have also increased the generalizability of our findings.

Nevertheless, this study has several limitations. Previous studies have found that vaccine hesitancy is complicated, time and location vary, and adherence-specific matter depends on the perceived behavioral nature of the community. ^31^^–^^33^ We conducted this study when the COVID-19 detection rate in the community was significantly lower than it has been, which may have influenced the perceived threat of the disease and the vaccine hesitancy rate. This study did not measure social and traditional media influences, which may have confounded the results. ^34^ Lastly, our questionnaire did not include questions specific to attitudes, beliefs, or mistrust about the vaccine.

## Conclusions

To ensure the optimum coverage of vaccines, the government, public health officials, and advocates should be prepared to address parental vaccine hesitancy to reach their target and establish programs to improve childhood COVID-19 vaccine literacy among parents. Availability of safety and efficacy data for COVID-19 vaccines for children in social and traditional media, community and healthcare centers, and mosques/temples would likely positively impact community dwellers’ attitudes toward childhood COVID-19 vaccination. Engaging community and religious leaders, family physicians, and trustworthy relatives should accelerate advocacy programs to reduce parental vaccine hesitancy for their children in the community.

## Data availability

### Underlying data

Open Science Framework: Parental coronavirus disease vaccine hesitancy for children in Bangladesh: a cross-sectional study. https://doi.org/10.17605/OSF.IO/43G5M ^35^

The project contains the following underlying data:

- Parental Vac Hesitancy F1000.sav (raw data from questionnaires)

### Extended data

Open Science Framework: Parental coronavirus disease vaccine hesitancy for children in Bangladesh: a cross-sectional study. https://doi.org/10.17605/OSF.IO/43G5M ^35^

The project contains the following extended data:

- Parental Vac Questionnaire.docx

### Reporting guidelines

Open Science Framework: Parental coronavirus disease vaccine hesitancy for children in Bangladesh: a cross-sectional study. https://doi.org/10.17605/OSF.IO/43G5M ^35^

This project contains the following reporting guidelines checklist:

- STROBE_checklist_Par_Vac_Hesi.docx

Data are available under the terms of the Creative Commons Attribution 4.0 International license (CC-BY 4.0).
