# Supplementary material for: Parental coronavirus disease vaccine hesitancy for children in Bangladesh: a cross-sectional study
Source: F1000Res. 2022 Mar 2;11:90. Originally published 2022 Jan 25. [Version 2] doi: 10.12688/f1000research.76181.2 (PMC8837808; doi:10.12688/f1000research.76181.2)
Supplement: Peer review history

# Peer review: Reviewer response for version 2

**DOI:** 10.5256/f1000research.121888.r125945

**Published:** 2022-03-16

**Authors:** F. M. Moinuddin

**Affiliations:** 1 Mayo Clinic Neuro-Informatics Laboratory, Mayo Clinic, Rochester, MN, USA

Revised version is satisfactory.

Is the work clearly and accurately presented and does it cite the current literature?

Yes

If applicable, is the statistical analysis and its interpretation appropriate?

Yes

Are all the source data underlying the results available to ensure full reproducibility?

Yes

Is the study design appropriate and is the work technically sound?

Yes

Are the conclusions drawn adequately supported by the results?

Yes

Are sufficient details of methods and analysis provided to allow replication by others?

Yes

Reviewer Expertise:

Methodology and structure

I confirm that I have read this submission and believe that I have an appropriate level of expertise to confirm that it is of an acceptable scientific standard.

# Peer review: Reviewer response for version 2

**DOI:** 10.5256/f1000research.121888.r125946

**Published:** 2022-03-14

**Authors:** Shakil Ahmed

**Affiliations:** 1 Maternal and Child Health Division, ICDDRB, Dhaka, Bangladesh

**Further comments:**

**Methods:**

*Study design and participants:*

 1. Do not use the "Approximately" term in the Methodology section. Please use the exact number of the parents.

Is the work clearly and accurately presented and does it cite the current literature?

Yes

If applicable, is the statistical analysis and its interpretation appropriate?

Yes

Are all the source data underlying the results available to ensure full reproducibility?

Yes

Is the study design appropriate and is the work technically sound?

Yes

Are the conclusions drawn adequately supported by the results?

Partly

Are sufficient details of methods and analysis provided to allow replication by others?

Partly

Reviewer Expertise:

Sexual and reproductive health, Maternal health, Child health, Non-communicable diseases, Pandemics, COVID-19, Gender research

I confirm that I have read this submission and believe that I have an appropriate level of expertise to confirm that it is of an acceptable scientific standard.

# Peer review: Reviewer response for version 2

**DOI:** 10.5256/f1000research.121888.r125944

**Published:** 2022-03-03

**Authors:** Dr. Manzur Kader

**Affiliations:** 1 Post doctoral researcher, Institute of Environmental Medicine, Karolinska Institutet, Stockholm, Sweden

No more comments.

Is the work clearly and accurately presented and does it cite the current literature?

Yes

If applicable, is the statistical analysis and its interpretation appropriate?

Yes

Are all the source data underlying the results available to ensure full reproducibility?

Yes

Is the study design appropriate and is the work technically sound?

Yes

Are the conclusions drawn adequately supported by the results?

Yes

Are sufficient details of methods and analysis provided to allow replication by others?

Yes

Reviewer Expertise:

Public health, Global Health, Epidemiology,

I confirm that I have read this submission and believe that I have an appropriate level of expertise to confirm that it is of an acceptable scientific standard.

# Peer review: Reviewer response for version 1

**DOI:** 10.5256/f1000research.80146.r120978

**Published:** 2022-02-11

**Authors:** Dr. Manzur Kader

**Affiliations:** 1 Post doctoral researcher, Institute of Environmental Medicine, Karolinska Institutet, Stockholm, Sweden

**Abstract**

- In methods, the Authors say “Predictors were identified using binary logistic regression analysis. Rephrase it like “Factors associated with vaccine hesitancy were identified using binary logistic regression analysis”. Because identifying predictors require a stronger study design than the cross-sectional study.
- Replace the word “face-to-face interviews” with an interview-administered questionnaire. Because “face-to-face interviews” is commonly used in a qualitative study
- Write the name of the questionnaire used to assess vaccine hesitancy

**Results**

 Results should be more specific:

- children's age (specify the age?), parent's age (specify the age?, religion (specify?), occupation (specify what occupation?), monthly household income (what level?), permanent address, living location (zone? Specify)
- Write “associated factors with parental vaccine hesitancy”, instead of “the independent predictors of parental vaccine hesitancy

**Introduction**

- Authors provide some data of prevalence of vaccine hesitancy, for example, the USA, and China (high-resourced countries).” Provide some similar examples from low-resourced countries if available as the study setting.
- Provide a clear justification of vaccine hesitancy, why this might happen? In relation to fear, safety, the effectiveness of vaccines? It’s not clear the setting of reference 13, specify it.
- Authors say,  “along with sociodemographic variabilities and religious beliefs….” Specify the sociodemographic variabilities.

**Methods**

- “completeness, accuracy, and internal consistency and secured them …..” Did you use any statistical test to assess such internal validity or reliability of the questionnaire? For example, to assess internal consistency requires Cronbach's alpha. Or did you asses it just by visual perception?  specify.
- Was the vaccine hesitancy questionnaire validated in a similar population like in Bangladesh? write some more about the questionnaire.

**Results**

- Write higher secondary, instead of  “College education”. Write BDT instead of “<৳”

**Discussion**

- Write a statement if the results agree with the hypothesis?

Is the work clearly and accurately presented and does it cite the current literature?

Yes

If applicable, is the statistical analysis and its interpretation appropriate?

Yes

Are all the source data underlying the results available to ensure full reproducibility?

Yes

Is the study design appropriate and is the work technically sound?

Yes

Are the conclusions drawn adequately supported by the results?

Yes

Are sufficient details of methods and analysis provided to allow replication by others?

Yes

Reviewer Expertise:

Public health, Global Health, Epidemiology,

I confirm that I have read this submission and believe that I have an appropriate level of expertise to confirm that it is of an acceptable scientific standard.

# Response

**Published:** 2022-02-27

**Authors:** Mohammad Ali

**Affiliations:** Uttara Adhunik Medical College, Bangladesh

Dear reviewer, thank you very much for your precise suggestions. We have revised our manuscript in light of your directions.

**Abstract:**

 Method

1. We relaced the word predictor by associated factors.
2. Most of the studies used a web-based data collection method in the pandemic time. However, we use the in-person interviews (face-to-face) method to get high-quality data.
3. The question was named as vaccine hesitancy questionnaire.

Result

1. & 2. We rewrote the result section.

**Main text:**

 Introduction:

1. Data regarding parental vaccine hesitancy in low and middle-income countries were scarce; thus, we could not add more references.
2. We amended the sentence.
3. Example added.

Method:

1. We can not extract Cronbach's alfa value for a questionnaire consisting of only one.
2. This questionnaire was used for a vaccine hesitancy study among adults in Bangladesh (Ali & Hossain, 2021).

Result:

1. The symbol is well accepted and used by the British Medical Journal.

Discussion:

  

1. The statement has been added in the discussion section.

References:

 Ali, M., & Hossain, A. (2021). What is the extent of COVID-19 vaccine hesitancy in Bangladesh? A cross-sectional rapid national survey. *BMJ Open*, *11*(8), e050303. https://doi.org/10.1136/bmjopen-2021-050303

# Peer review: Reviewer response for version 1

**DOI:** 10.5256/f1000research.80146.r121413

**Published:** 2022-02-09

**Authors:** F. M. Moinuddin

**Affiliations:** 1 Mayo Clinic Neuro-Informatics Laboratory, Mayo Clinic, Rochester, MN, USA

The paper presented intends to show the prevalence of parental vaccine hesitancy in Bangladesh. They found that vaccine hesitation varied based on sociodemographic characteristics, religion, behavior, and perceived COVID-19 threat. The paper may be useful for the policymaker to identify the obstacle in the mass vaccination program. The paper is well written and well-planned. The methodology is acceptable. However, the following comments are recommended:

1. The authors should change p=0.000 to <0.001 for better understanding in Table 1.
2. Please add a zero before a decimal point in Table 1.
3. The COVID vaccine is recommended for students aged between 12 and 17 years in Bangladesh. However, the study participant who had children below 12 years is more than 78% of the total participants. The author should mention the justification for including this group in their discussion.

Is the work clearly and accurately presented and does it cite the current literature?

Yes

If applicable, is the statistical analysis and its interpretation appropriate?

Yes

Are all the source data underlying the results available to ensure full reproducibility?

Yes

Is the study design appropriate and is the work technically sound?

Yes

Are the conclusions drawn adequately supported by the results?

Yes

Are sufficient details of methods and analysis provided to allow replication by others?

Yes

Reviewer Expertise:

Methodology and structure

I confirm that I have read this submission and believe that I have an appropriate level of expertise to confirm that it is of an acceptable scientific standard.

# Response

**Published:** 2022-02-27

**Authors:** Mohammad Ali

**Affiliations:** Uttara Adhunik Medical College, Bangladesh

Dear reviewer, thank you very much for your review and insight. We have revised our paper according to your suggestions.

  

1. We replaced 0.000 by <0.001.
2. In table 1, zero has been added before the decimal point.
3. You are right; we get most data for children under 12. However, the vaccine is being approved for all the pediatric population (0-<18 years). This study will be useful when the COVID-19 vaccine is approved for children under 12 years.

# Peer review: Reviewer response for version 1

**DOI:** 10.5256/f1000research.80146.r120976

**Published:** 2022-02-08

**Authors:** Shakil Ahmed

**Affiliations:** 1 Maternal and Child Health Division, ICDDRB, Dhaka, Bangladesh

**Abstract: **

- The abstract requires a more precise statement in each section, i.e., Background, Methods, Results, and Conclusion.
- The Methods are not clear. As the first line of the “Methods” indicates, the parents of children <18 years were interviewed between October 10 – October 31, 2021. However, the following line points out that the parents aged ≥18 years underwent face-to-face interviews. At first glance, it is not easy to understand the study population of this study.
- The key independent predictors of parental vaccine hesitancy should contain more numbers, i.e., odds ratio (OR) and 95% CI’s upper and lower values. Otherwise, it will not be easy for the readers to grasp the primary findings of this study just by reading the abstract.

**Introduction:**

- It would be more standard if the author would use the word “more than/greater than/similar terms” instead of using the sign “>” in the introduction.
- There are some grammatical errors in the introduction section (especially in the articles). Please correct.
- It’s not clear why vaccine hesitancy research is essential for Bangladesh. A clear justification is required with proper reference and evidence.
- Why are the parents aged ≥18 years important? Is there any evidence?

**Methods:**

**Study design and participants:**

- Please clarify what do you mean by Parents aged ≥18 years? Whether it is the father, aged ≥18 years or the mother ≥18 years or both? What did you do if one of the parents was aged <18 years?
- More clear inclusion and exclusion criteria should be mentioned in the manuscript.
- What language did you use in the vaccine hesitance questionnaire to collect the data?
- It would be great for the general readers if the authors could add the sample size formula in the manuscript
- Did the authors validate the vaccine hesitancy questionnaire for the Bangladeshi population as they mentioned they were using a previously employed vaccine hesitancy questionnaire? Is there any cultural validation evidence?

**Study questionnaire:**

- It would be great if the authors could attach the vaccine hesitancy questionnaire as  supplementary documents for the ease of the readers both English and Bengali (if any), which they have used to collect data
- The authors did not add any funding information. Please add.

**Sampling technique and data collection:**

 How did the authors choose the marketplaces, shopping malls, waiting rooms of large hospitals, diagnostic centres, bus and rail stations randomly? Did they acquire any list? If yes, then from where did you acquire the list? Please provide evidence (if there’s any)

**Statistical analysis:**

- How do you dichotomize the four responses to vaccine hesitancy? Is there any evidence or previous research which have done the same? Please provide the proper evidence.
- Age, Number of children, Educational qualification, and monthly household income are ordinal variables. What analysis did you perform for these variables to compare with the vaccine hesitancy?
- Why did you consider all the variables in the logistic regression model? Please explain.

**Results:**

- As you have collected data from the eight divisions of Bangladesh, it would be logical if the author could show the difference in divisional vaccine hesitancy of Bangladesh. Then it would be easier for the policymaker to emphasize the efforts where it is more required

Parents and children characteristics (Table 1):

- Please use the term “lost a family member” instead of using “lost a loved one.”
- Please do not refer the readers as – “Details of the responses to the questions regarding the likelihood of children or family members' infection by COVID-19 and the level of concern about children or family members' disease in the next year are shown”. The author should describe the findings.

Results of descriptive analysis (Table 1):

- The author should not use the content of the outcome variable as the independent variables and use it in the analysis, such as – “Perceived likelihood of children or family members' infection in the next year”, “Level of concern about children or family members' infection in the next year”. Please correct.
- For a better understanding, please mention the meaning of the sign “৳” as “Bangladeshi Taka/BDT/Taka/similar term” in the footnote of the table or the manuscript.

Results of regression analysis (Table 2):

- Please mention the comparison group in the table-2’s header. Otherwise, it is hard to interpret for the readers.
- The author should not use the content of the outcome variable as the independent variables and use it in the analysis, such as – “Perceived likelihood of children or family members' infection in the next year”, “Level of concern about children or family members' infection in the next year”. Please correct.
- The religion variable’s result might be overestimated. The author considered the lowest number of population group “Other”, as the reference group. This variable should be categorized into “Muslim and Non-muslim”.

**Strengths and Limitations**

- What did the author mean by 1%, non-muslim parents? Here, the groups should be mentioned as written in Table 1.

**Conclusions**

- What are the recommendations for the policymakers, and how do the policymakers should address the recommendations are not clear. Please elaborate.

Is the work clearly and accurately presented and does it cite the current literature?

Yes

If applicable, is the statistical analysis and its interpretation appropriate?

Yes

Are all the source data underlying the results available to ensure full reproducibility?

Yes

Is the study design appropriate and is the work technically sound?

Yes

Are the conclusions drawn adequately supported by the results?

Partly

Are sufficient details of methods and analysis provided to allow replication by others?

Partly

Reviewer Expertise:

Sexual and reproductive health, Maternal health, Child health, Non-communicable diseases, Pandemics, COVID-19, Gender research

I confirm that I have read this submission and believe that I have an appropriate level of expertise to confirm that it is of an acceptable scientific standard, however I have significant reservations, as outlined above.

# Response

**Published:** 2022-02-27

**Authors:** Mohammad Ali

**Affiliations:** Uttara Adhunik Medical College, Bangladesh

Dear reviewer,  thank you very much for your time and insight. We have amended our manuscript based on your directions. Please find the point-by-point solution to the problems.

**Abstract:**

1. We have amended the abstract according to your suggestion.
2. The correction has been made in the method section. We replaced < by below where appropriate.
3. The result section has been rewritten. However, we can not add AOR and CI due to word restrictions of the abstract.

**Main text:**

 Introduction:

1. We replaced symbols with words where applicable.
2. Please be assured that this manuscript is grammatical-error-free; however, we rechecked and made amendments where needed.
3. We amended the justification of the study.
4. The age of marriage in Bangladesh is 18 years and above, thus, we have taken data only from adult parents.

Methods:

 Study design

1. We exclude parents <18 years. We clarified inclusion and exclusion criteria.
2. We use the Bangla language, clearly mentioned in the method section.
3. The sample size formula has been described clearly; please find the formula with appropriate references.
4. This is a questionnaire consisting of one question. British Medical Journal has published data using this questionnaire in Bangladesh (Ali & Hossain, 2021).

Questionnaire

1. Please find the questionnaire in the supplementary section.
2. Funding information has been added; find the information at the upper end of the article.

Sampling technique

1. We clearly described that the list had been collected from the division's website.

Statistical analysis

1. Yes, we followed previously published studies when categorising the vaccine intention (Ali & Hossain, 2021; Khubchandani et al., 2021).
2. We used descriptive analysis for categorical variables.
3. The final model has included significant variables found in the descriptive analysis. Find the information in the statistical analysis section.

Results

1. Our aim was not to compare the hesitancy considering division; rather, we compared the zones of Bangladesh. Future studies should compare the prevalence of COVID-19 vaccine hesitancy according to the divisions. 
2. We replaced the loved one with a family member. Thanks for the suggestion.
3. To reduce the words, we refer a few to the table.
4. We use perceived threat as the independent variable, which is one of our core findings. This variable has been used in previous studies as an independent variable.
5. Symbol of Bangladeshi taka is well accepted any used previously by British Medical Journal. We kept the symbol.
6. Mentioned.
7. We used the variable as it is at the core of vaccine hesitancy. We discussed the implications in the discussion section.
8. We have shown the diversity of religions in the study that clearly reflects the general Bangladeshi population. However, when we take this variable in regression, we combined Christian and Buddhists (total 21 samples).
9. We corrected the error. Thank you for your meticulous observation.

Conclusion

1. We rewrote the conclusion section.

Finally, we would like to thank you again for your time and dedication when reviewing the paper. Your suggestion made the paper improve than before.

 References:

 Ali, M., & Hossain, A. (2021). What is the extent of COVID-19 vaccine hesitancy in Bangladesh? A cross-sectional rapid national survey. *BMJ Open*, *11*(8), e050303. https://doi.org/10.1136/bmjopen-2021-050303

 Khubchandani, J., Sharma, S., Price, J. H., Wiblishauser, M. J., Sharma, M., & Webb, F. J. (2021). COVID-19 Vaccination Hesitancy in the United States: A Rapid National Assessment. *Journal of Community Health*, 1–8. https://doi.org/10.1007/s10900-020-00958-x
